# Targeting tubular epithelial cell metabolism to halt renal fibrosis: current evidence and future directions

**DOI:** 10.3389/fcell.2026.1849072

**Published:** 2026-07-02

**Authors:** Wenke Liu, Xing Zhao

**Affiliations:** 1 Department of Thoracic Surgery, the First Affiliated Hospital of China Medical University, Shenyang, Liaoning, China; 2 Department of Pediatrics, the First Affiliated Hospital of China Medical University, Shenyang, Liaoning, China

**Keywords:** chronic kidney disease, fatty acid oxidation, glycolysis, metabolic reprogramming, mitochondrial dysfunction, renal fibrosis

## Abstract

Renal fibrosis is a common pathological endpoint of chronic kidney disease, characterized by excessive deposition of extracellular matrix and loss of functional nephrons. Emerging evidence highlights that profound metabolic reprogramming is a hallmark and a fundamental driver of renal fibrosis. Alterations in the central metabolic pathways, including glycolysis, mitochondrial dysfunction and lipid metabolism in tubular epithelial cells promote tubular epithelial cell dysfunction and sustain a pro-fibrotic inflammatory microenvironment. Besides, the metabolic microenvironment, particularly characterized by hypoxia and oxidative stress, plays a critical role in the pathogenesis and progression of renal fibrosis, and targeting the metabolic microenvironment has also emerged as a promising anti-fibrotic strategy. In this review, we summarized the current knowledge and explored the potential of targeting metabolic enzymes or signaling pathways involved in key metabolic pathways may represent a promising strategy to attenuate renal fibrosis. Furthermore, we discussed the contribution of the metabolic microenvironment, including hypoxia and oxidative stress, to the pathogenesis of renal fibrosis.

## Introduction

Chronic kidney disease (CKD) is a global health burden. Renal fibrosis, marked by fibroblast proliferation and extracellular matrix (ECM) accumulation, is essential for CKD progression to end-stage renal disease (Romagnani et al., 2017). Given the current lack of effective treatments for renal fibrosis, discovering strategies to ameliorate or reverse renal fibrosis is of great clinical significance. While renal fibrosis is a multicellular process, tubular epithelial cells (TECs) serve as both the sensors of injury and key drivers of fibrogenesis through metabolic reprogramming, cytokine secretion, and partial epithelial-mesenchymal transition (Liu et al., 2021). Therefore, targeting TEC-centric metabolism represents a rational and increasingly validated therapeutic strategy. Emerging evidence has established a close relationship between metabolic reprogramming and the pathogenesis of renal fibrosis (Liu et al., 2021; Miguel et al., 2025). Although the kidney is not generally considered a metabolic organ, it requires energy to regulate fluid volumes and osmolality, mediate filtration and excretion, maintain fluid and electrolyte balance. Notably, aberrant metabolic reprogramming can induce renal fibrosis, while renal injury reciprocally perturbs metabolic homeostasis. Accordingly, metabolic dysfunction and renal fibrosis engage in a bidirectional, mutually exacerbating relationship, which leads to a vicious cycle for the progression of renal fibrosis (Wei et al., 2023).

**FIGURE 1:**
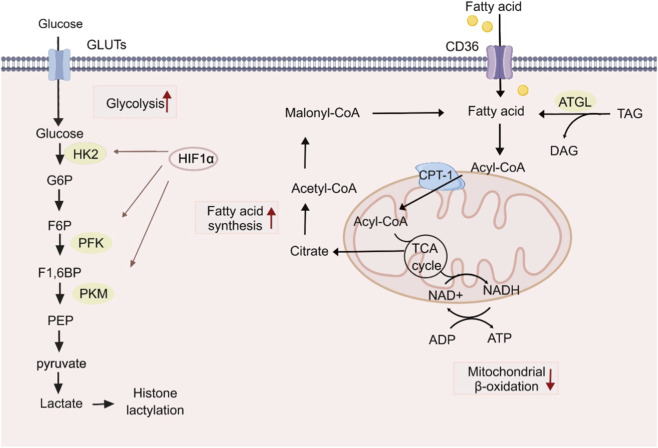
The mechanisms underlying metabolic reprogramming in tubular epithelial cells during renal fibrosis. G6P, Glucose-6-phosphate; F6P, Fructose-6-phosphate; F1,6BP, Fructose-1,6-bisphosphate; PEP, Phosphoenolpyruvate; TCA, tricarboxylic acid; TAG, Triacylglycerol; DAG, Diacylglycerol.

In this review, we present a discussion of recent advances regarding the mechanisms underlying metabolic reprogramming in renal fibrosis, with a focus on the regulatory mechanisms of core metabolic pathways, including glycolysis, mitochondrial dysfunction, and lipid metabolism within TECs (Table 1). The potential of targeting metabolic enzymes or signaling pathways involved in key metabolic pathways as novel therapeutic strategies for renal fibrosis has also been revealed (Figure 1). Furthermore, we also focused on the dynamic regulatory role of the microenvironment in the progression of renal fibrosis. Through this review, we aim to establish a foundation for the development of novel targeted therapeutic approaches for renal fibrosis.

**TABLE 1:** Targeting metabolic enzymes or regulators involved in key metabolic pathways.

Target	Effect	Model	Treatment	References
Timp2	Timp2 knockout alleviates renal fibrosis by preserving mitochondrial dynamics	-	cyclopamine	Pang et al. (2025)
LTBP4	LTBP4 potentially protects against renal fibrosis *via* mitochondrial homeostasis	-	knockdown	Su et al. (2023)
HK2	HK2 promoted PKM2/LDHA axis and lactate-driven renal fibrosis	UUO and adenine (Ade) - induced CKD mouse models	saikosaponin C	Deng et al. (2025)
PFKM	PFKP overexpression promoted TGF-β1-induced glycolysis	UUO models	isorhamnetin	Yang et al. (2023)
PFKP	miR-21a-5p delivered by MSC-Exos inhibited the expression of PFKM, thereby attenuating glycolytic flux	UUO models	MSC-Exos	Xu et al. (2022)
PFKFB3	PFKFB3-mediated tubular glycolytic reprogramming markedly enhanced histone lactylation, particularly at H4K12	IRI mouse model	knockout	Wang et al. (2024)
GPR87	GPR87 accelerated glycolysis and mitochondrial injury by YAP-hexokinase-2 signaling	UUO models	knockout	Cui et al. (2022)
ATF4	ATF4 transcriptionally upregulated HK2 to facilitate renal fibrosis through HK2 mediated glycolytic activation	UUO models	knockdown	Feng et al. (2025)
KLF5	KLF5 recognized the promotor of cdh1 and inhibited its transcription to accelerate EMT.	Db/db mice	Knockdown or ML264	Zhang et al. (2024a)
CPT1A	Cpt1a-knockin mice exhibited reduced expression of fibrotic markers, attenuated proinflammatory responses, diminished epithelial cell damage, and decreased macrophage infiltration	-	Gain-of-function	Miguel et al. (2021)
ACSM3	ACSM3 deficiency disrupted MCFA metabolism and impaired mitochondrial homeostasis	-	(AAV)-mediated ACSM3 overexpression	Li et al. (2025a)
CB2	CB2 activated β-catenin signaling, resulting in the suppression of PPARα/PGC-1α axis to decrease FAO functions and lipid droplet formation in tubular cells	FAN mice	knockout	Zhou et al. (2024)
TST	TST downregulation diminished the S-sulfhydration of very long-chain specific acyl-CoA dehydrogenase, leading to mitochondrial FAO dysfunction	-	sodium thiosulfate or TST overexpression	Zhang et al. (2024b)
STAT6	STAT6 activation led to lipid accumulation and downregulation of FAO-related genes	UUO or high-fat diet-fed mice	Tubule-specific Stat6 deletion, STAT6 inhibition, or Stat6 knockdown	Li et al. (2022)
Twist1	Twist1 overexpression impaired FAO, increased intracellular lipid droplet accumulation, mitochondrial dysfunction, and elevated production of pro-fibrotic factors	-	pharmacological inhibition of Twist1 with Harmine	Liu et al. (2022)
Pyruvate carboxylase	PC deficiency promoted mitochondrial damage, mtDNA leakage, and activation of the cGAS-STING pathway	-	knockout	Huang et al. (2025)
TFAM	TFAM deficiency aberrant promoted mtDNA packaging and cytosolic translocation, activated the cGAS–STING pathway	​	knockout	Chung et al. (2019)
IGFBP7	IGFBP7 enhanced PKM2 dimerization and nuclear translocation to promote lipid production and renal fibrosis	-	salmeterol	Yu et al. (2025)
KIM-1	KIM-1 mediated uptake of palmitic acid (PA)-bound albumin in proximal tubular, leading to enhanced tubule injury with DNA damage, cell-cycle arrest and renal fibrosis	-	TW-37	Mori et al. (2021)

## Metabolic reprogramming in TECs

### Glycolysis and TECs

The shift from oxidative phosphorylation to glycolysis has emerged as a key driver of renal fibrosis. In unilateral ureteral obstruction (UUO) mice, glycolysis levels were markedly elevated, whereas inhibition of TEC glycolysis using mesenchymal stem cell-derived exosomes (MSC-Exos) significantly attenuated renal fibrosis (Xu et al., 2022). Numerous studies have demonstrated that the expression of glycolytic enzymes is altered in patients with renal fibrosis. Increased expression of glycolytic enzymes, including hexokinase-2 (HK2) and Pyruvate Kinase M2 (PKM2), was observed in renal tissues with fibrosis and correlated with the progression of renal fibrosis. Treatment of HK-2 cells with the glycolytic activator TEPP-46 significantly upregulated the expression of fibrosis markers (Huang et al., 2025). In UUO and adenine-induced CKD mouse models, saikosaponin C disrupted the HK2/PKM2/LDHA axis, reducing lactate-driven renal fibrosis (Deng et al., 2025). Phosphofructokinase (PFK), another rate-limiting glycolytic enzyme, has three subtypes: the liver type (PFKL), muscle type (PFKM), and platelet type (PFKP), catalyzing the irreversible conversion of fructose-6-phosphate into fructose-1,6-bisphosphate. Yang et al. found that PFKP was critical for the metabolic switch to glycolysis in TECs during fibrosis development. In UUO-models, blocking PFKP with isorhamnetin notably ameliorated TEC-elevated glycolysis and renal fibrosis (Yang et al., 2023). MSC-Exos significantly ameliorated UUO-induced renal fibrosis by inhibiting PFKM-mediated glycolysis in TECs. Mechanistically, miR-21a-5p delivered by MSC-Exos inhibited the expression of PFKM, thereby attenuating glycolytic flux in TECs and the progression of renal fibrosis (Xu et al., 2022). Besides, tubule-specific GPR87 deletion dramatically ameliorated renal fibrosis in UUO mice. Mechanistically, GPR87 accelerated glycolysis and mitochondrial injury by YAP-HK2 signaling, thereby promoting renal fibrosis (Cui et al., 2022). These findings suggest that targeting key glycolytic enzymes and regulators is essential for the treatment of renal fibrosis.

In addition to its role in cellular metabolism, glycolytic reprogramming promotes renal fibrosis *via* non-canonical pathways. Specifically, lactate, the glycolytic byproduct, can modulate gene expression *via* epigenetic modifications through histone lactylation, thereby facilitating fibrogenesis in TECs (Wei et al., 2025). It has been found that reducing the lactate levels significantly inhibited EMT progression and improved renal tubular fibrosis in diabetic kidney disease (Zhang et al., 2024b). Histone lactylation, particularly H3K14la, contributed to EMT process by facilitating KLF5 expression. Moreover, KLF5 recognized the promotor of cdh1 and inhibited its transcription to accelerate EMT. Additionally, specific knockdown and pharmacological inhibition of KLF5 diminished EMT development and attenuated renal fibrosis (Zhang et al., 2024b). Similarly, lactate derived from PFKFB3-mediated tubular glycolytic reprogramming markedly enhanced histone lactylation, particularly at H4K12. This modification was enriched at the promoters of NF-κB signaling genes, including Ikbkb, Rela, and Relb, thereby activating their transcription and facilitating the inflammatory response. In the ischemia-reperfusion injury (IRI) mouse model, TEC-specific deletion of PFKFB3 markedly decreased lactate levels in the kidney, attenuated renal fibrosis, and protected renal function (Wang et al., 2024). Taken together, these findings reveal that the central role of glycolytic reprogramming in renal fibrosis, indicating that targeting glycolysis with specific inhibitors may hold therapeutic potential. Critically, the overactivated glycolysis may not act in isolation. Lactate accumulation and intracellular acidification directly impair mitochondrial function and oxidative phosphorylation (Gan et al., 2026). Thus, glycolysis may function as the upstream trigger that initiates the cascade of metabolic failure in renal fibrosis.

### Mitochondria and TECs

Mitochondria are pivotal intracellular organelles that play crucial roles in regulating energy production, oxidative stress, calcium homeostasis, and apoptosis (Zhang et al., 2024a). TECs are characterized by an abundance of mitochondria that render them highly susceptible to pathological circumstances. Mitochondrial homeostasis in TECs is governed by a complex regulatory network. Peroxisome proliferator-activated receptor gamma coactivator-1α (PGC-1α) serves as the master regulator of mitochondrial biogenesis and function, transactivating genes involved in oxidative phosphorylation and antioxidant defense (Abu Shelbayeh et al., 2023). Mitochondrial transcription factor A (TFAM) is essential for mtDNA maintenance and replication (Liu et al., 2024). Dynamin-related protein 1 (DRP1) regulates mitochondrial fission, and its dysregulation leads to mitochondrial fragmentation and dysfunction (Jin et al., 2021). This regulatory network coordinately regulates mitochondrial dysfunction in fibrotic TECs. Upon mitochondrial dysfunction, TECs undergo a phenotypic switch towards a secretory state, releasing pro-inflammatory mediators, including cytokines, chemokines, growth factors, reactive oxygen species, that promotes the inflammatory microenvironment and drives renal fibrosis progression (Li et al., 2024; Ma et al., 2026).

Analysis of renal tissues in patients and mice models with fibrosis revealed a marked mitochondrial defect in TECs, characterized by loss of TFAM. Renal fibrosis was observed in mice with tubule-specific deletion of *Tfam*. In TFAM knockout mice, aberrant mitochondrial DNA (mtDNA) packaging led to its cytosolic translocation, activating the cytosolic cGAS-STING DNA sensing pathway and subsequently inducing cytokine expression and immune cell recruitment. Notably, genetic ablation of STING mitigated kidney fibrosis in mouse models of CKD, establishing that TFAM sequesters mtDNA to limit inflammation and subsequent fibrosis (Chung et al., 2019). Pyruvate carboxylase (PC) has been observed a marked reduction of PC expression in TECs from both mouse and human fibrotic kidneys. Conditional deletion of PC in TECs aggravated UUO-induced renal fibrosis. Mechanistically, PC deficiency compromised the stability of sulfide:quinone oxidoreductase (SQOR), leading to mitochondrial damage, mitochondrial DNA (mtDNA) leakage, and subsequent activation of the cGAS-STING signaling pathway. This cascade promoted glycolytic reprogramming and ultimately led to renal fibrosis (Huang et al., 2025). Besides, Tubule-specific Timp2 knockout alleviates renal fibrosis by preserving mitochondrial dynamics. Mechanistically, Timp2 interacts with the extracellular domain of syndecan-4 (Sdc4) in an autocrine manner, thereby activating the Hedgehog (Hh) signaling pathway. Treatment with cyclopamine partially reversed Timp2 overexpression-induced mitochondrial dysfunction, suppressed PFKFB3-mediated glycolysis, and reduced collagen deposition. (Pang et al., 2025). Latent transforming growth factor beta binding protein 4 (LTBP4) potentially protects against renal fibrosis *via* mitochondrial homeostasis during the transition from acute kidney injury (AKI) to CKD. Loss of LTBP4 led to enhanced ROS production and inflammatory responses under ischemic/hypoxic conditions, which were ameliorated by DRP1 inhibition. Therefore, targeting LTBP4-regulated DRP1-dependent mitochondrial fission may offer novel therapeutic strategies for AKI and progressive renal fibrosis (Su et al., 2023). In summary, mitochondrial homeostasis is critically linked to renal fibrosis, and targeting mitochondrial dysfunction offers a potential therapeutic avenue.

### Lipid metabolism and TECs

**FIGURE 2:**
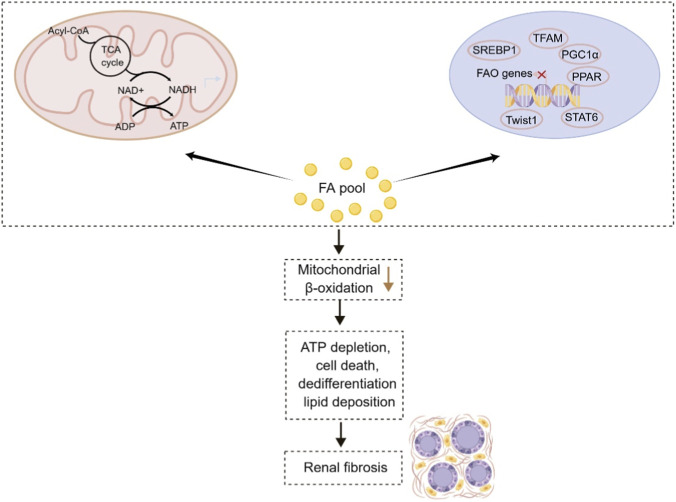
The mechanisms underlying fatty acid oxidation in tubular epithelial cells during renal fibrosis.

Lipid metabolism is closely associated with renal fibrosis, and dysregulation of lipid metabolism is commonly observed in patients with CKD (Chen et al., 2022). A direct metabolic consequence of mitochondrial dysfunction is the impairment of fatty acid oxidation (FAO), which subsequently leads to ATP depletion and intracellular lipid droplet accumulation (Wedan et al., 2024). Consequently, the dysfunctional mitochondrion serves as a pivotal hub, bridging the initial glycolytic state with a more profound state of lipid reprogramming, thereby establishing a pathological foundation for lipid abnormalities. FAO is a central metabolic pathway in mitochondria that catabolizes long-chain fatty acids to generate acetyl-CoA, which is subsequently utilized through the tricarboxylic acid (TCA) cycle and oxidative phosphorylation to produce ATP. Defective FAO in renal tubular cells represents a key pathological feature of renal fibrosis (Figure 2). It has been found that reduced expression of FAO enzymes was observed in a large cohort of patients with renal fibrosis (Kang et al., 2015). FAO inhibition in TECs induced ATP depletion, cell death, dedifferentiation, and lipid deposition, whereas genetic or pharmacological restoration of FAO protected mice from renal fibrosis (Kang et al., 2015).

Carnitine palmitoyltransferase 1A (CPT1A) is a mitochondrial outer membrane enzyme that catalyzes the rate-limiting step of long-chain FAO. It has been found that Cpt1a-knockin mice exhibited reduced expression of fibrotic markers, attenuated pro-inflammatory responses, diminished epithelial cell damage, and decreased macrophage infiltration. Gain-of-function in FAO restored oxidative metabolism, preserved mitochondrial mass, and enhanced bioenergetics, resulting in increased FAO and ATP levels. Notably, analysis of human CKD patients revealed decreased CPT1A levels and accumulation of short- and medium-chain acylcarnitines, indicative of impaired FAO. Therefore, strategies targeting gain-of-function in FAO may represent a therapeutic approach to inhibit renal fibrosis in CKD (Miguel et al., 2021). Adipose triglyceride lipase (ATGL) is a key enzyme governing lipid hydrolysis. Pharmacological inhibition of ATGL with atglistatin exacerbated lipid accumulation, suppressed FAO activity, and impaired mitochondrial function, while concomitantly increasing oxidative stress and apoptosis, ultimately leading to aggravated renal fibrosis (Li et al., 2025b). Cannabinoid Receptor 2 (CB2) activates β-catenin signaling, resulting in the suppression of PPARα/PGC-1α axis. This decreased FAO functions and led to lipid droplet formation in tubular cells. CB2 deficiency effectively mitigated FAO dysfunction, lipid deposition and uremic toxins accumulation in FAN mice, consequently retarding renal fibrosis. Additionally, inhibition to β-catenin or PPARα activation could greatly inhibit lipid accumulation and fibrogenesis induced by CB2 (Zhou et al., 2024). Thiosulfate sulfurtransferase (TST) is a mitochondrial enzyme critical for sulfur transfer, and its expression is reduced in patients with renal fibrosis. Genetic deficiency of TST exacerbated tubular damage in both diabetic and renal fibrosis mouse models, whereas pharmacological intervention with sodium thiosulfate or TST overexpression protected against high glucose-induced tubular injury. Mechanistic investigations revealed that TST downregulation diminished the S-sulfhydration of very long-chain specific acyl-CoA dehydrogenase, leading to mitochondrial FAO dysfunction (Zhang et al., 2024c). Signal transducer and activator of transcription 6 (STAT6) regulates tubular lipid metabolism in renal fibrosis. In UUO or high-fat diet-fed mice, STAT6 activation was correlated with lipid accumulation and downregulation of FAO-related genes. Tubule-specific Stat6 deletion, STAT6 inhibition, or Stat6 knockdown attenuated lipid accumulation and fibrosis by enhancing FAO (Li et al., 2022). TWIST1 is another key transcription factor that regulates lipid metabolism in renal fibrosis. Overexpression of Twist1 suppressed PGC-1α transcription, leading to reduced expression of FAO-associated genes, including PPARα, CPT1, and ACOX1. In human proximal tubular HK-2 cells, this resulted in impaired FAO, increased intracellular lipid droplet accumulation, mitochondrial dysfunction, and elevated production of pro-fibrotic factors. Conversely, Twist1 knockout mice with renal injury exhibited increased PGC-1α expression, restored FAO, and attenuated progression of renal fibrosis. Notably, pharmacological inhibition of Twist1 with Harmine reduced lipid accumulation and restored FAO both *in vitro* and *in vivo* (Liu et al., 2022). Acyl-Coenzyme A synthetase medium-chain family member 3 (ACSM3), a key enzyme in medium-chain fatty acid (MCFA) metabolism, has been implicated in renal fibrosis. Both systemic and tubule-specific Acsm3 knockout exacerbated renal fibrosis, whereas adeno-associated virus (AAV)-mediated ACSM3 overexpression alleviated fibrosis in mice. Mechanistically, ACSM3 deficiency disrupted MCFA metabolism and impaired mitochondrial homeostasis. Notably, we identified that dodecanoic acid (C12:0), a fatty acid primarily utilized by ACSM3 in the kidney, inhibited renal fibrosis (Li et al., 2025a). These findings highlight the therapeutic importance of targeting key enzymes or regulators involved in fatty acid metabolism in renal fibrosis.

Lipid synthesis and uptake also play critical roles in the pathogenesis of renal fibrosis. Aberrant lipid metabolism, characterized by enhanced *de novo* lipogenesis and increased lipid uptake, is increasingly recognized as a key driver of renal fibrosis (Lee et al., 2024). Excessive lipid accumulation in renal cells leads to lipotoxicity, oxidative stress, and activation of pro-fibrotic signaling pathways, including TGF-β/Smad and NLRP3 inflammasome (Yang et al., 2017). These metabolic disturbances collectively promote epithelial-mesenchymal transition, fibroblast activation, and ECM deposition, ultimately culminating in progressive renal fibrosis. IGFBP7 bound to pyruvate kinase M2 (PKM2) to promote the acetylation of PKM2 at the K433 site, thereby enhancing PKM2 dimerization and nuclear translocation, and subsequently accelerating lipid production and renal fibrosis *via* SREBP1-dependent mechanisms. Notably, through drug screening, we identified salmeterol as an IGFBP7 antagonist that effectively reduced fibrosis (Yu et al., 2025). Kidney injury molecule-1 (KIM-1) mediates uptake of palmitic acid (PA)-bound albumin in proximal tubular, leading to enhanced tubule injury with DNA damage, cell-cycle arrest and renal fibrosis. TW-37 has been identified as a small molecule inhibitor of KIM-1-mediated PA-albumin uptake and showed *in vivo* in a kidney injury model in mice that it attenuated renal fibrosis (Mori et al., 2021). These findings highlight the therapeutic importance of targeting fatty acid synthesis and uptake in renal fibrosis.

### Hypoxia and ROS as the primary upstream integrators

In fibrotic kidneys, persistent injury creates a microenvironment characterized by hypoxia and excessive reactive oxygen species (ROS) production. Rather than acting as independent stressors, hypoxia and ROS function as central integrators that simultaneously engage multiple metabolic pathways, orchestrating a coordinated metabolic reprogramming of TECs.

Hypoxia, as an inherent pathophysiological characteristic of CKD, functions as a critical mediator in the occurrence and development of renal fibrosis (Naas et al., 2023; Wang et al., 2022). The hypoxic state in the kidney primarily results from microvascular loss, reduced oxygen diffusion, and cellular metabolic abnormalities (Liu et al., 2017). Under hypoxic exposure, enhanced glycolysis was observed in TECs to promote the progression of renal fibrosis (Jiang et al., 2023). Hypoxia-inducible factor 1 (HIF-1) is a heterodimeric nuclear transcription factor that serves as a master regulator of cellular adaptation to low oxygen level (Semenza, 2001). The inducible HIF-1α subunit functions as the master transcriptional integrator. Chronic hypoxia stabilizes HIF-1α, which simultaneously activates the expression of target genes involved in glycolysis (GLUT1, HK2, PFKFB3, LDHA, and PKM2) and lactate transport (Ke Q and Costa M, 2006). Notably, HIF-1α directly binds to the regulatory region of the *Ppargc1a* promoter, suppressing PGC-1α expression, a key regulator of mitochondrial biogenesis and FAO (Park et al., 2025). This HIF-1α-mediated suppression of PGC-1α creates a feed-forward loop that perpetuates mitochondrial dysfunction and metabolic reprogramming. This creates a vicious cycle in which impaired oxidative metabolism leads to greater reliance on glycolysis, which in turn drives lactate accumulation and subsequent epigenetic reprogramming, leading to persistent HIF-1α stabilization. Autophagy related 5 (ATG5) has been found to promote renal fibrosis by stabilizing HIF-1α through its mediation of HSP90 binding, which in turn induced glycolytic activation. This metabolic reprogramming subsequently induced mitochondrial fission and inflammatory responses, contributing to the progression of renal fibrosis (Hu et al., 2025). Diosmin exerted its protective effects by binding to HIF-1α and blocking its nuclear translocation, leading to reduced FABP4 transcription and subsequent attenuation of inflammation, collagen deposition, and ferroptosis (Zhao et al., 2025). However, HIF-1α plays a dual role in the progression of renal fibrosis. HIF-1α could protect against renal fibrosis by orchestrating BNIP3-mediated mitophagy, which in turn decreased ROS and inhibited NLRP3 inflammasome activation (Li et al., 2023a). These studies suggest that a more comprehensive investigation into the role of HIF-1α in the progression of renal fibrosis under hypoxic conditions is of considerable value for identifying potential therapeutic targets to suppress renal fibrosis.

The oxidatively stressed extracellular microenvironment is an active driver of renal fibrosis (Jiménez-Uribe et al., 2021). It orchestrates fibroblast activation, ECM remodeling, metabolic dysfunction, and inflammatory responses, establishing a vicious cycle that leads to progressive renal fibrosis (Su et al., 2019; Gyurászová et al., 2020). Oxidative stress results from an imbalance between increased ROS production and reduced antioxidant defense capacity (Sies, 2015). The best-characterized cellular antioxidants are the glutathione peroxidases (GPXs), a family of enzymes with peroxidase activity to protect cells from oxidative damage (Pei et al., 2023). It has been found that depletion of glutathione peroxidase-3 (GPX3) in TECs following renal injury constitutes a key determinant creating an oxidatively stressed extracellular microenvironment, which in turn drives interstitial fibroblast activation and proliferation. GPX3-depleted extracellular microenvironment spontaneously induced NADPH oxidase-4 (NOX4) expression and ROS production in renal fibroblasts, leading to activation and proliferation of renal fibroblasts. Activation of NOX4 by advanced oxidation protein products (AOPPs) phenocopied the effects of GPX3 loss, increasing ROS production, stimulating fibroblast activation and proliferation, and activating the protein kinase C-α (PKCα)/mitogen-activated protein kinase (MAPK)/signal transducer and activator of transcription 3 (STAT3) signaling pathway. Silencing NOX4 or inhibiting MAPK with small-molecule inhibitors suppressed fibroblast activation and proliferation. Collectively, GPX3 loss orchestrates an oxidatively stressed extracellular microenvironment that promotes fibroblast activation and proliferation through a cascade of signal transduction (Li et al., 2023b). Besides, Li et al. found that GPX3 knockdown also led to upregulation of NADPH oxidase 2, enhanced renal ROS generation, and activation of p38 mitogen-activated protein kinase. Conversely, overexpression of exogenous GPX3 attenuated kidney fibrosis and suppressed NADPH oxidase-2 expression as well as p38 mitogen-activated protein kinase activity (Li et al., 2023c). These findings indicate that oxidative stress constitutes a critical component of the fibrogenic microenvironment and provide novel insights into the molecular composition of the fibrogenic microenvironment.

Critically, these metabolic pathways interact through multiple positive feedback loops. For instance, lactate produced by sustained glycolysis promotes TGF-β1 expression *via* histone lactylation. TGF-β1 in turn suppresses PGC-1α and PPARα, deepening FAO inhibition and perpetuating mitochondrial dysfunction (Xiang et al., 2025). Besides, mitochondrial ROS, generated from impaired electron transport chain complexes I and III under hypoxic conditions, functions as a critical integrator that simultaneously damages multiple components of oxidative metabolism. ROS directly inactivates TCA cycle enzymes such as aconitase and impairs CPT1A activity, further compromising FAO capacity (Xu et al., 2025).

### Clinical translation of metabolic-targeted therapies in renal fibrosis

In recent years, metabolic-targeted therapies, including glycolysis inhibitors (2-deoxyglucose), FAO activators (etomoxir), and AMPK agonists (metformin) have shown promising anti-fibrotic effects in preclinical models of renal fibrosis (Ajibade et al., 2024; Chen et al., 2022; Gao et al., 2025; Wei et al., 2023). However, translating these agents into clinical practice for renal fibrosis requires careful evaluation of pharmacological and safety parameters that are often inadequately addressed in preclinical studies. Importantly, several metabolic modulators are already in clinical trials or approved for kidney diseases, offering valuable lessons for future development.

Despite their efficacy in preclinical models, many metabolic modulators exhibit significant off-target and on-target toxicities that raise concerns for long-term use in CKD patients. For example, bardoxolone methyl, an NRF2 activator that improves mitochondrial function, reached Phase 3 trials for diabetic kidney disease but was associated with fluid retention and cardiovascular events, likely due to off-target effects in endothelial cells (Li et al., 2025b). Despite the favorable safety profile of metformin in mild-to-moderate CKD, its use in CKD remains controversial because of concerns about increased lactic acidosis risk (Inzucchi et al., 2014; Patiño-Cardona et al., 2026). The bioavailability of metabolic-targeted agents in the fibrotic kidney is another major translational hurdle, which can be profoundly altered in the setting of CKD due to gut dysbiosis, delayed gastric emptying, and altered drug transporters. For instance, niclosamide, a glycolysis inhibitor with potent anti-fibrotic activity in preclinical studies, has extremely poor oral bioavailability, necessitating novel nanoparticle or prodrug formulations currently under investigation (Rehman et al., 2018; Jug et al., 2024). In summary, while metabolic-targeted therapies offer an attractive approach for treating renal fibrosis, clinical translation will significantly depend on overcoming challenges related to drug safety, bioavailability, route of administration, and adverse effect profiles. Future research should focus on integrating pharmacokinetic assessments, developing kidney-selective delivery systems, and conducting long-term safety studies.

## Conclusion

Metabolic reprogramming is recognized as a critical driver of renal fibrosis. The shift from oxidative metabolism toward glycolytic and anabolic pathways across diverse renal cell types establishes a cycle that fuels ECM production, chronic inflammation, and cellular dysfunction. Elucidation of this metabolic landscape in the fibrotic kidney opens new avenues for biomarker discovery and therapeutic intervention. Pharmacological modulation of these aberrant metabolic pathways holds considerable promise for the development of treatments capable of reversing CKD progression.
